# Tri-functional platform for construction of modular antibody fragments for *in vivo*^18^F-PET or NIRF molecular imaging†

**DOI:** 10.1039/c9sc05007h

**Published:** 2020-01-07

**Authors:** Raymond F. Gamache, Kirstin A. Zettlitz, Wen-Ting K. Tsai, Jeffrey Collins, Anna M. Wu, Jennifer M. Murphy

**Affiliations:** Department of Chemistry and Biochemistry, University of California Los Angeles CA 90095 USA; Department of Molecular and Medical Pharmacology and Crump Institute for Molecular Imaging, David Geffen School of Medicine, University of California Los Angeles CA 90095 USA jmmurphy@mednet.ucla.edu

## Abstract

Positron emission tomography (PET) molecular imaging is a powerful tool for interrogating physiological and biochemical processes to understand the biology of disease and advance therapeutic developments. Near-infrared fluorescence (NIRF) optical imaging has become increasingly popular for intraoperative staging to enable cellular resolution imaging of tumor margins during surgical resection. In addition, engineered antibody fragments have emerged as promising molecular imaging agents given their exquisite target selectivity, rapid systemic clearance and site-selective chemical modification. We report a tri-functional platform for construction of a modular antibody fragment that can rapidly be labeled with radionuclides or fluorophores for PET or NIRF molecular imaging of prostate stem cell antigen (PSCA).

## Abstract 2

To provide a universal approach towards the targeted delivery of PET and optical imaging agents, we have developed a tri-functional platform (TFP) for the facile construction of modular, target-specific tracers.

## Graphical abstract

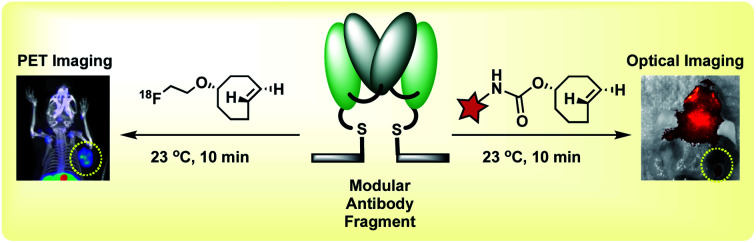

## Introduction

Molecular imaging techniques such as positron emission tomography (PET) and near-infrared fluorescence (NIRF) optical imaging are highly valuable tools for diagnosis, staging and therapy monitoring of disease. These modalities offer complimentary data, with PET enabling whole-body functional information about disease localization and potential metastasis while the optical component provides fluorescent-guided intraoperative staging.^1^ A significant advantage of PET in the cancer space is the noninvasive tumor detection and localization of an expressed biomarker throughout all tissues of the body.^2^ Visualization of antigen-positive cells *via* optical imaging during surgery can assist surgeons to maximize resection of primary and metastatic cancer cells, minimize resection of healthy tissue, limit potential recurrence and improve overall survival rate for cancer patients.^3^

Antibody-based PET (immunoPET) is a versatile platform for the development of molecularly targeted, sensitive and quantitative imaging of cell-surface protein expression *in vivo*.^4^ Advances in antibody engineering techniques to reduce immunogenicity, modify pharmacokinetics and improve clearance have facilitated the development and translation of antibody fragments to be utilized as molecular imaging probes.^5^ Recombinant antibody fragments called cys-diabodies (cysteine-modified scFv dimers; 50 kDa) contain a C-terminal cysteine that forms a disulfide bridge to stabilize dimerization which can be preferentially reduced to unveil free thiols for well-defined, site-specific conjugation.^5*b*,6^ Importantly, the cysteine modification is located away from the antigen-binding site to preserve immunoreactivity of the protein conjugate. These constructs are particularly powerful imaging agents due to their small size, high tumor uptake, thiol-specific conjugation and fast blood clearance which results in high tumor to background ratios within 2 hours of probe injection.^6*b*^ Labeled with positron-emitting radionuclides (^18^F, ^64^Cu, ^124^I and ^89^Zr), recombinant diabody/cys-diabody constructs have been employed as immunoPET probes for a variety of tumor types including breast, pancreatic, prostate and colorectal cancers as well as for tumor-infiltrating CD8^+^ T cells.^5*b*,6*a*,7^ In addition, fluorescently labeled antibody fragments have been evaluated *in vivo* for their potential use as targeted optical imaging probes in xenograft models.^8^

To provide a universal approach towards the targeted delivery of PET and optical imaging agents, we have developed a tri-functional platform (TFP) for the construction of modular cys-diabodies. This novel platform rapidly yields a site-specific bioconjugate with improved pharmacokinetic properties that can be readily labeled *via* a highly efficient (4+2) cycloaddition reaction to incorporate either fluorine-18 or a near-infrared fluorescent dye. This robust strategy leverages the tetrazine-*trans*-cyclooctene ligation which has been gaining interest for the selective introduction of fluorine-18 into biologically relevant molecules.^9^ The remarkable speed of this ligation is well suited for ^18^F-labeling of proteins, where the short radioactive half-life and stringent conditions demand the need for rapid, efficient chemistry.^10^

For proof-of-concept, a cys-diabody against prostate stem cell antigen (PSCA) was conjugated to the tri-functional platform and immunoPET and optical imaging were performed to access whole-body biodistribution as well as tumor targeting potential in mice carrying prostate cancer xenografts. This model demonstrates not only the capability of the platform to afford molecular imaging tracers that provide high-contrast images against PSCA-positive cells, but also reveals the potential of this approach to provide *in vivo* information on cell-surface protein expression for any target-specific construct.

## Results and discussion

### Tri-functional platform design and synthesis

Exploiting strain-energy to facilitate molecular reactivity has been established as a highly effective strategy, prompting the discovery and applications of promising bioorthogonal reactions.^11^ The inherent nature of the inverse electron demand Diels–Alder (IEDDA) cycloaddition^12^ between tetrazine and *trans*-cyclooctene – catalyst-free, extremely rapid, highly selective, modular, robust – make it an attractive reaction for protein labeling applications and provided the rationale to incorporate it into the tri-functional platform design and construction.^13^

The maleimide synthon is a powerful tool for site-specific bioconjugation, and perhaps the most common functional group for selective modification of proteins. Fast kinetics and remarkably high chemoselectivity towards cysteine residues account for its persistent popularity in protein labeling. Despite susceptibility of the succinimidyl thioether linkage to hydrolysis and retro-Michael reactions, the half-life of a maleimide-protein linkage is ∼60 h *in vivo* which is considerably longer than the 3–7 h circulating half-life of a cys-diabody.^14,5*b*^ The rapid pharmacokinetics of cys-diabodies permit bioconjugation *via* maleimide-based synthons and is well established in molecular imaging applications.^7*e*,8*b*,27*a*^

**Fig. 1:**
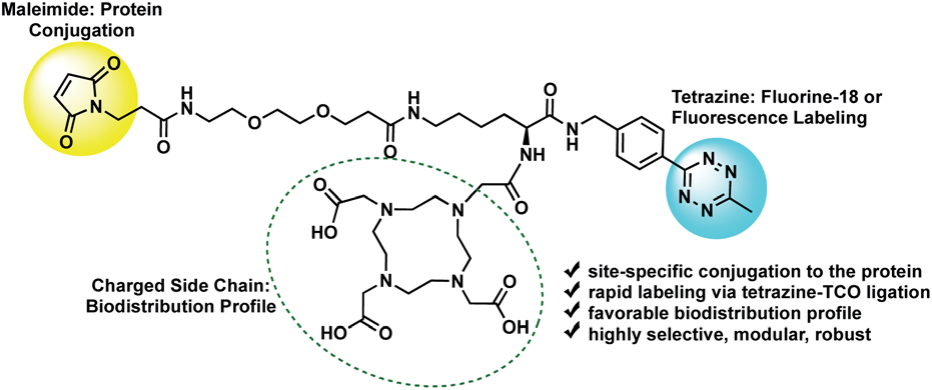
Tri-functional platform for construction of modular antibody fragments.

The tri-functional platform consists of three key moieties: (1) a maleimide for site-specific conjugation to the cys-diabody *via* a succinimidyl thioether linkage; (2) a tetrazine for rapid labeling of the immunoconjugate *via* a (4+2) cycloaddition reaction; and (3) a hydrophilic side chain to improve the *in vivo* biodistribution profile and favor renal clearance (Fig. 1). Traditional labeling methods require optimization of the protein conjugation, purification and characterization steps each time the labeling is conducted, which may lead to inconsistent batches or variability in purity and immunoreactivity. The modular strategy affords flexibility by providing a stock of cys-diabody, with a favorable biodistribution profile, that can readily be labeled *via* rapid (4+2) cycloaddition with a broad range of TCO-modified tags without the need for further optimization of reaction conditions. Ultimately, the power of this approach lies in its modularity; it enhances the versatility of a single cys-diabody, potentially of any desired specificity, without compromising affinity to its molecular target, and facilitates facile labeling with radionuclides and fluorophores.

A critical challenge in the development of molecular imaging agents is the image contrast between signal and background. Radiolabeled antibodies and their fragments can display exquisite target affinity, with predictable kinetics and clearance routes *in vivo*. However, imaging is also impacted by the catabolism and the clearance/retention of subsequent radiometabolites, which are strongly influenced by properties such as lipophilicity and charge. Much has been learned from pharmacokinetics and biodistribution studies on radiolabeled peptides, which have been modulated by chemical moieties such as aspartate residues,^15^ histidine/glutamic conjugates,^16^ succinic acid,^17^ glycosylation^18^ and the metal chelator DOTA.^19^ Increasing negative charge or incorporating chemical groups such as these has resulted in increased plasma clearance^20^ and diverted tracer or metabolite clearance in favor of renal over hepatobiliary.^18*a*,19*b*^

Previously, our group reported a dual-modality linker for conjugation to a cys-diabody for imaging PSCA expression.^21^ With a molecular weight of ∼53 kDa, below the threshold for first-pass renal clearance, the initial clearance route for the bioconjugate was *via* the kidneys. Radiometabolites were reabsorbed and ultimately routed through the hepatobiliary system, resulting in extended blood pool activity and retention in the liver, kidneys and gastrointestinal tract (GI), presumably due to high lipophilicity of tracer radiometabolites.^21^ Size, structure and charge distribution are key properties that influence renal retention of radiolabeled metabolites.^22^ As a result of this study, the tri-functional platform was designed to preserve kidney retention of radiometabolites, reduce liver and GI activity and provide a clean background in the lower abdomen by incorporating an anionic moiety to decrease lipophilicity. We chose to incorporate a DOTA for its ease of synthetic installation and its potential for chelation of therapeutic radioisotopes towards the future development of theranostic tools. The rationale for our platform design is strengthened by a recent report that biodistribution of an ^18^F-labeled peptide tracer could be significantly modulated by installation of a metal-free DOTA appendage.^19*b*^

**Scheme 1:**
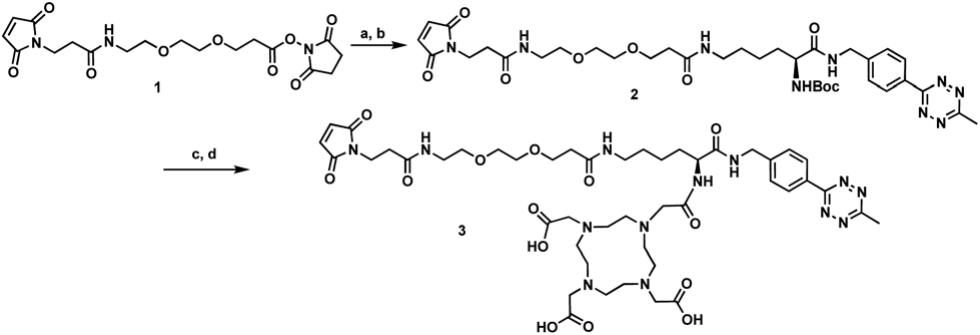
Synthesis of the tri-functional platform **3**. Reagents and conditions: (a) *N*-Boc-l-lysine, i-Pr_2_NEt, DMF, 23 °C, 16 h, 78%; (b) 4-(6-methyl-1,2,4,5-tetrazin-3-yl)benzyl amine, HATU, i-Pr_2_NEt, DMF, 23 °C, 36 h, 71%; (c) 4 M HCl/dioxane, 23 °C, 6 h, 40%; (d) DOTA-NHS, Et_3_N, DMF, 23 °C, 16 h, 49%. HATU = 1-[bis(dimethylamino)methylene]-1*H*-1,2,3-triazolo[4,5-*b*]pyridinium 3-oxid hexafluorophosphate; DOTA-NHS = 1,4,7,10-tetraazacyclododecane-1,4,7,10-tetraacetic acid mono-*N*-hydroxysuccinimide ester.

The tri-functional platform **3** was prepared in four steps starting from commercially available *N*-boc-l-lysine (Scheme 1). PEGylated succinimidyl ester **1** was readily conjugated to *N*-boc-l-lysine to furnish the maleimide–lysine conjugate. We chose to employ the 3-benzylamino-6-methyl-1,2,4,5-tetrazine for its improved chemical stability at the sacrifice of slightly slower cycloaddition kinetics.^23^ Thus, the benzylamino tetrazine was synthesized as previously reported^24^ and subjected to peptide coupling with the maleimide–lysine conjugate to furnish intermediate **2** in good yield. Acid mediated deprotection followed by ligation with commercial DOTA-NHS ester afforded the desired tri-functional platform **3**.

### Modular antibody fragment construction

**Fig. 2:**
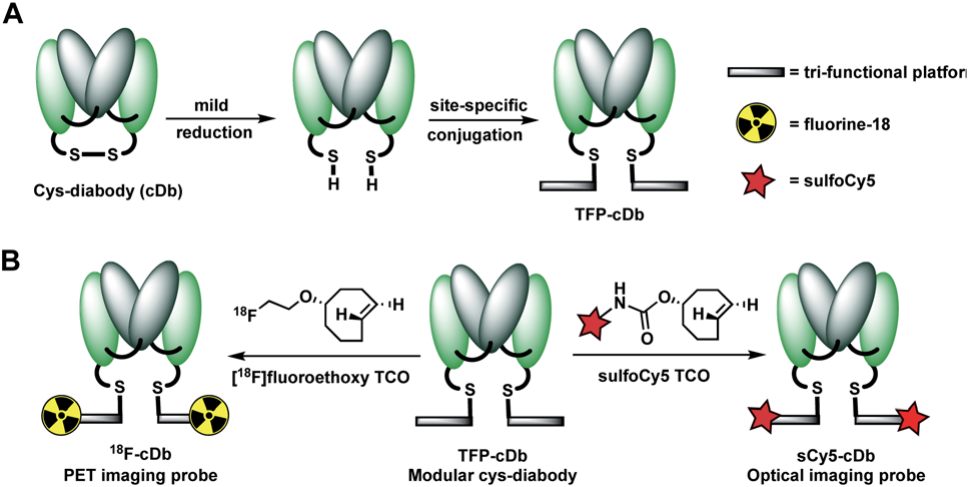
(A) Construction of modular antibody fragment TFP-cDb. (B) Labeling with fluorine-18 or sulfoCy5 for PET or NIR optical imaging.

Prostate stem cell antigen (PSCA) is a cell surface protein expressed by prostate, pancreatic and bladder cancers with low expression in normal tissues and no expression in bone or lymph nodes, primary sites of cancer metastasis.^25^ Nearly all prostate cancers express high levels of PSCA and higher expression levels correlate with poor prognosis and metastatic disease, thus making it an attractive diagnostic and therapeutic biomarker for these cancer types.^26^ Previously, we developed humanized anti-PSCA A2 cys-diabody fragments and demonstrated their use as molecular imaging probes to visualize prostate and pancreatic tumors in both transgenic and xenograft mouse models.^7*h*,8*b*,27^ In our current study, the anti-PSCA A2 (cDb) was site-specifically conjugated to the tri-functional platform (**3**) and utilized for PET and NIR optical imaging. The construction process starts with mild reduction of the cys-diabody to cleave the disulfide bridge and provide two free thiols (Fig. 2A).

Following reduction, site-specific conjugation to the tri-functional platform was achieved *via* a Michael addition between the sulfhydryl and maleimide group, producing the modular cys-diabody TFP-cDb. Excess reagents were separated from the protein conjugate using a gel filtration column and the modular cys-diabody TFP-cDb was analyzed by polyacrylamide gel electrophoresis (SDS-PAGE) and size exclusion chromatography (SEC) (Fig. S3†). To afford ^18^F-cDb or sCy5-cDb, purified TFP-cDb efficiently underwent a Diels–Alder cycloaddition at room temperature with either [^18^F]fluoroethoxy TCO or sulfoCy5 TCO, respectively (Fig. 2B). The modularity of this approach allows for the creation of a common stock of immunoconjugate, TFP-cDb, that can directly be applied to either PET or optical imaging.

### *In vivo* molecular imaging

**Fig. 3:**
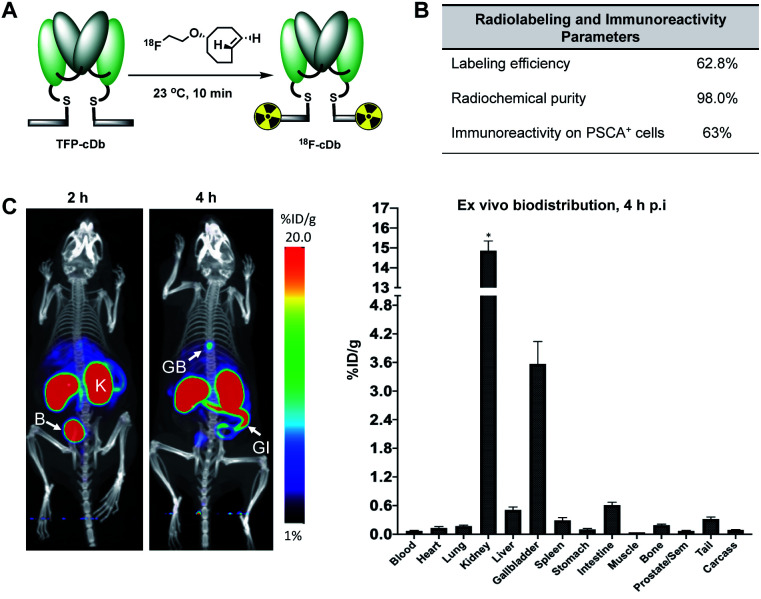
Radiolabeling and *in vivo* microPET/CT imaging. (A) Radiolabeling schematic for the (4+2) cycloaddition reaction between TFP-cDb and [^18^F]fluoroethoxy TCO to generate ^18^F-cDb. (B) Radiolabeling and immunoreactivity characteristics of ^18^F-cDb. (C) ^18^F-cDb was injected i.v. into male nude mice (*n* = 4) and PET images were acquired. Representative PET/CT maximum intensity projection (MIP) images in one mouse at 2 and 4 h p.i. *Ex vivo* biodistribution at 4 h p.i. *count rate maximum of gamma counter was exceeded. B, bladder; K, kidney; GB, gallbladder; GI, gastrointestinal tract.

Synthesis of [^18^F]fluoroethoxy TCO was automated on the ELIXYS FLEX/CHEM radiochemical synthesizer (Sofie Biosciences) as described previously.^28^ For ^18^F-labeling, reformulated [^18^F]fluoroethoxy TCO (37 MBq) was added to TFP-cDb (100 μg in 100 μL PBS, pH 7.4) and the mixture was incubated for 10 minutes at room temperature (Fig. 3A). Purification by micro-spin size exclusion spin column afforded ^18^F-cDb with a specific activity of 0.17 MBq μg^−1^. Radiolabeling efficiency and radiochemical purity of ^18^F-cDb were measured by instant thin layer chromatography (ITLC) and determined to be 63% and 98% respectively. The immunoreactive fraction (63%) was well within the previously reported range for A2 cys-diabody based immunoPET tracers (40–80%) (Fig. 3B).^21^

**Fig. 4:**
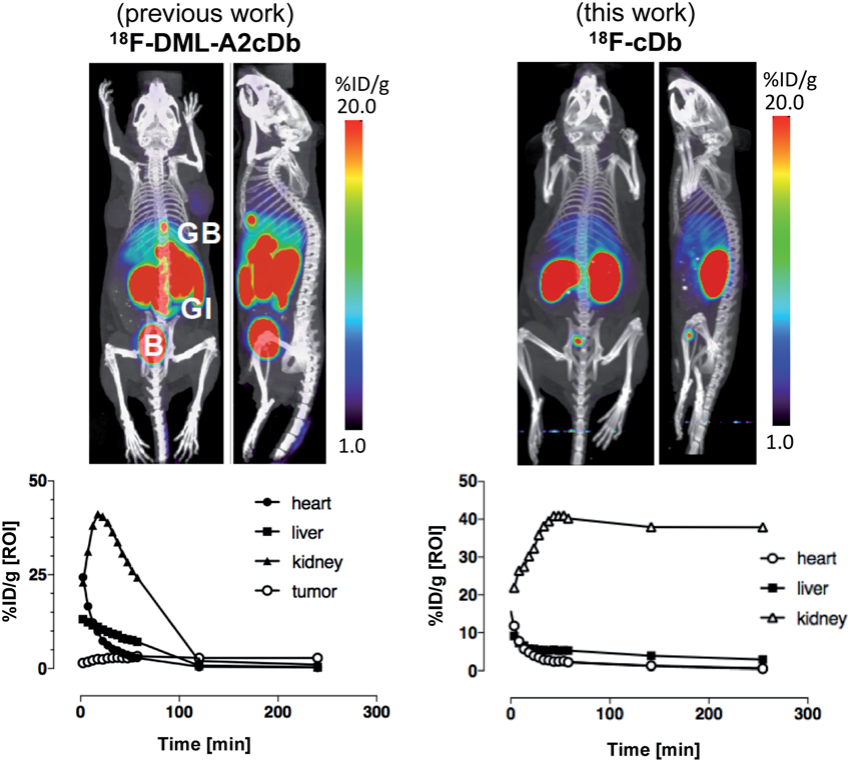
ImmunoPET imaging and ROI analysis comparison between the dual-modality linker and tri-functional platform. Representative PET/CT MIP images in nude mice at 1 h p.i. of ^18^F-DML-A2cDb or ^18^F-cDb and quantitative ROI analysis of heart, liver and kidney. B, bladder; GB, gallbladder; GI, gastrointestinal tract. Adapted from research originally published in JNM. Zettlitz KA, Waldmann CM *et al.* A dual-modality linker enables site-specific conjugation of antibody fragments for ^18^F-immunoPET and fluorescence imaging. *J Nucl Med.*, 2019, DOI: 10.2967/jnumed.118.223560. ©SNMMI.

Many PET probes are metabolized *via* the hepatobiliary route, resulting in high liver signal which hinders the ability to visualize liver metastasis. In order to maintain low background signal in the liver and generate high contrast images, renal clearance is highly desirable for PET molecular imaging probes. To evaluate *in vivo* imaging and biodistribution, ^18^F-cDb (8.5 μg, 1.3 MBq) was injected *via* the tail vein into male nude mice (*n* = 4) and microPET/CT scans were acquired. Fig. 3C shows PET imaging and *ex vivo* biodistribution analysis which confirm that ^18^F-cDb demonstrated rapid clearance from circulation within 2 h followed by renal metabolism and retention of radiometabolites in the kidneys, enabling high contrast images within a few hours after probe injection (see also Fig. S4†). In comparison to our previously reported construct,^21^ the observed hepatobiliary clearance of radiometabolites was considerably decreased, as reflected by 3.6% ID g^−1^ activity in gallbladder and 0.51% ID g^−1^ activity in liver (Fig. 3C and Table S1†). A direct comparison between the previous construct and the current tri-functional platform is depicted in Fig. 4. Different biodistribution profiles are clearly defined within 1 h post tracer injection and distinct improvements with the DOTA-containing platform are further emphasized by the ROI analysis (Fig. 4). For the previous construct, there is initial renal clearance (evident in the kidney ROI on the left), followed by release and subsequent hepatobiliary clearance of radiometabolites, resulting in high liver uptake and retention of radioactivity in the GI tract, which can obscure visualization of metastases. The charged, hydrophilic side chain of the tri-functional platform ensures that, following initial renal clearance, there is retention of radiometabolites as indicated by significantly reduced liver signal in the PET/CT image (Fig. 4, right panel) as well as the consistent 40% ID g^−1^ kidney uptake in the ROI analysis. This resulted in lower non-specific background and an unobstructed view of the pelvic region that would allow for detection of prostate cancer. Importantly, a 22-fold reduction of radiometabolite retention in the GI was observed and the absence of bone uptake confirmed metabolic stability of ^18^F-cDb towards [^18^F]fluoride hydrolysis and release.

**Fig. 5:**
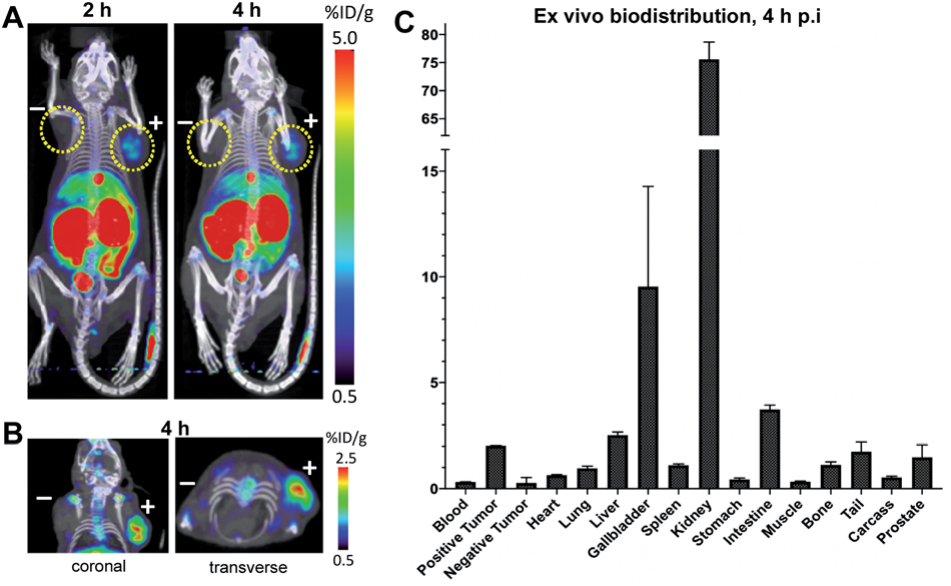
*In vivo* targeting of PSCA-positive tumors. (A) Male nude mice (*n* = 4) bearing PSCA-positive (right shoulder) and PSCA-negative (left shoulder) subcutaneous xenografts were administered ^18^F-cDb and microPET/CT images were acquired at 2 and 4 h p.i. Representative images in one mouse are shown as MIP/CT overlays. (B) Representative images in one mouse at 4 h p.i. are shown as 3 mm sections. (C) *Ex vivo* biodistribution at 4 h p.i. of ^18^F-cDb.

In order to evaluate the tumor targeting capability of ^18^F-cDb, an imaging study was performed in a mouse model of prostate cancer. Male nude mice (*n* = 4) bearing PSCA-positive (right shoulder) and PSCA-negative (left shoulder) subcutaneous xenografts were intravenously injected with ^18^F-cDb (10 μg, 1.85 MBq) and imaged at 2 and 4 h post injection (p.i.) (Fig. 5). At 4 h, the PET/CT images and *ex vivo* biodistribution analysis showed ∼8-fold higher probe uptake in the antigen-expressing tumor compared to the control tumor (Fig. 5 and Table S2†). Representative transverse and coronal PET/CT images provided clear tumor visualization (Fig. 5B).

These results independently confirm the influence of a DOTA moiety to reduce GI tract accumulation and to redirect clearance to the kidneys over liver, which was recently demonstrated by Roxin *et al.* using a VLA-4 radiopeptide.^19*b*^ Likewise, in the case of the radiopeptide study, a DOTA moiety reduced GI accumulation by 10-fold to 4.5% ID g^−1^ in tumor-bearing mice.^19*b*^ Analogous findings for both peptide and small protein-based imaging tracers reinforces the critical influence of hydrophilic, anionic chemical moieties, such as DOTA, on tracer biodistribution and performance. Additionally, in a radionuclide therapy application of our construct, long retention times of radiometabolites causing renal toxicity could be counteracted by infusion of basic amino acids (arginine, lysine) or polygelines (*e.g.* Gelofusine®) to block tubular reabsorption.^22*a*,29^

**Fig. 6:**
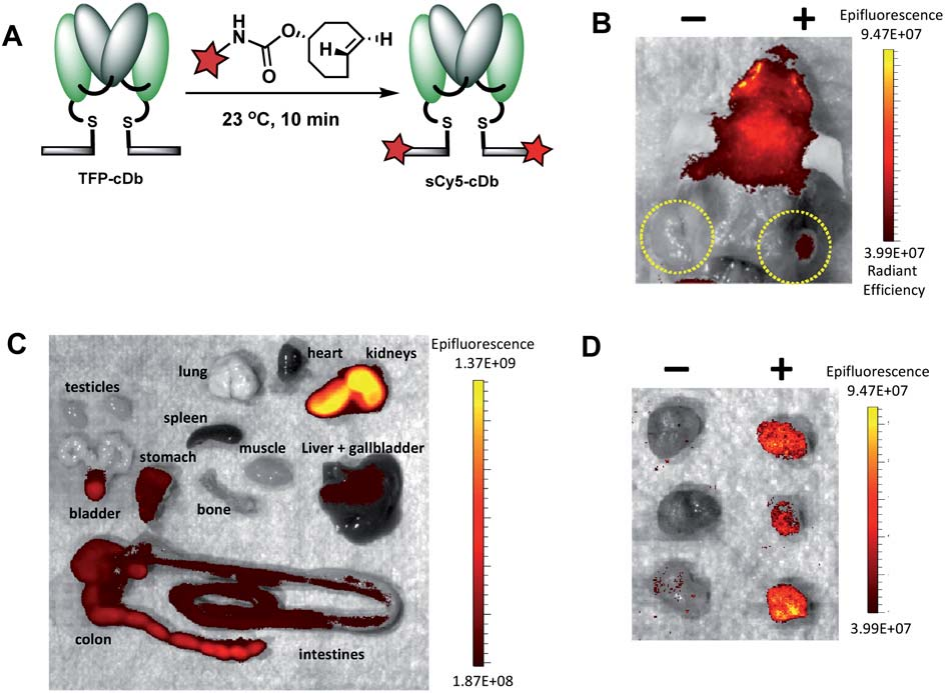
Fluorescence labeling and *in vivo* optical imaging of PSCA-expressing tumors. (A) Fluorescence labeling schematic for the (4+2) cycloaddition reaction between TFP-cDb and sCy5-TCO to generate sCy5-cDb. (B) Male nude mice (*n* = 3) bearing PSCA-positive (right shoulder) and PSCA-negative (left shoulder) subcutaneous xenografts were administered 20 μg sCy5-cDb. At 4 h p.i. mice were euthanized and optical images were acquired with the skin removed. (C) *Ex vivo* optical images of collected organs at 4 h p.i. to assess tracer and metabolite clearance (kidneys, bladder) and autofluorescence (liver, stomach, intestines). (D) Optical images of excised tumors confirm PSCA-specific fluorescent signals were detected with high positive : negative tumor contrast.

Fluorescence image-guided surgery allows for real-time tumor delineation. Optical imaging agents that use fluorescence to identify cancerous cells offers the potential advantage of intraoperative imaging, ultimately improving surgical margins and reducing incidence of cancer recurrence. To evaluate our platform for utility in image-guided surgery, the modular cys-diabody was labeled with a near infrared fluorescent dye, sulfo-cyanine 5. SulfoCy5 TCO (20 mM in DMF) was added to TFP-cDb (150 μg in 100 μL PBS, pH 7.4) at room temperature and, after 10 minutes, the (4+2) cycloadduct sCy5-cDb was obtained (Fig. 6A). SDS-PAGE and SEC confirmed the purity and integrity of the labeled protein (Fig. S5†).

Male nude mice (*n* = 3) bearing human prostate cancer xenografts, each with PSCA-positive cells implanted in the right shoulder and PSCA-negative cells implanted in the left shoulder, were used for the *in vivo* optical imaging experiment. Reformulated sCy5-cDb (20 μg) was injected *via* tail vein and mice were sacrificed 4 h after probe injection. Optical imaging performed post mortem with skin removed around the tumors allowed ready visualization of the antigen-positive tumors (Fig. 6B). Considerable autofluorescence from the skin results in high signal from the head (Fig. 6B). *Ex vivo* analysis of major organs revealed strong autofluorescence from the stomach and intestines and to a lesser extent the liver and spleen (Fig. 6C). As expected, renal clearance of sCy5-cDb resulted in high fluorescence signal in the kidneys. Notably, strong fluorescence signal was detected in the PSCA-positive tumors with negligible nonspecific uptake in PSCA-negative tumors (Fig. 6D). sCy5-cDb demonstrated excellent specificity for PSCA-expressing cells, providing high positive to negative signal contrast in the tumors and validating its potential utility for intraoperative staging. Alternative NIR fluorescent dyes, such as IRDye800CW or ICG, have been explored due to low autofluorescence, high spatial resolution and initial clinical success,^30^ and can be adapted to the current modular platform to enable rapid optimization of fluorescent dyes to identify the ideal fluorophore to utilize, which may be dependent on multiple variables. Taken together, this study demonstrates that the tri-functional platform can efficiently afford modular immunoconjugates for PET and optical imaging that maintain favorable *in vivo* biodistribution profiles and high affinity to the cell-surface protein of interest.

## Conclusions

In summary, we have synthesized a tri-functional platform and constructed a modular antibody fragment that targets PSCA, a well-established prostate and pancreatic cancer biomarker. Using the robust and rapid inverse electron demand Diels–Alder reaction between tetrazine and *trans*-cyclooctene, site-specific protein labeling was successfully accomplished to rapidly afford two molecular imaging constructs. ImmunoPET and NIRF optical imaging in mice bearing PSCA-positive subcutaneous tumors confirmed *in vivo* targeting and revealed specific tracer uptake and remarkable contrast in antigen-positive tumors. As envisioned, the DOTA moiety of the tri-functional platform favorably influenced ^18^F-cDb biodistribution and metabolite clearance to significantly diminish non-specific GI uptake. We anticipate that our approach will provide a straightforward path towards the development and clinical translation of immunoPET and optical imaging tracers based on antibody fragments of desired specificity.

## Conflicts of interest

A. M. Wu is a shareholder and consultant to ImaginAb, Inc.

## Supplementary Material

SC-011-C9SC05007H-s001
