# Supplementary material for: Tri-functional platform for construction of modular antibody fragments for in vivo18F-PET or NIRF molecular imaging
Source: Chem Sci. 2020 Jan 7;11(7):1832–8. doi: 10.1039/c9sc05007h (PMC8148382; doi:10.1039/c9sc05007h)
Supplement: SC-011-C9SC05007H-s001 [file SC-011-C9SC05007H-s001.pdf]

Electronic Supplementary Material (ESI) for Chemical Science.
This journal is © The Royal Society of Chemistry 2020

Supplementary Information

Tri-functional platform for construction of modular antibody fragments for in vivo ^{18}F-PET/NIRF molecular imaging

Raymond F. Gamache,[a,‡] Kirstin A. Zettlitz,[b,‡] Wen-Ting K Tsai,[b] Jeffrey Collins,[b] Anna M. Wu[b] and Jennifer M. Murphy*[b]

[a]Department of Chemistry and Biochemistry, [b]Department of Molecular and Medical Pharmacology and Crump Institute for Molecular Imaging, David Geffen School of Medicine

University of California Los Angeles

Los Angeles, California 90095, United States

Table of Contents

1. Materials and Methods……………………………………………………………………...S3

2. Experimental Data……………………………………………………………………… S4

2.1 Synthesis and characterization of the tri-functional platform (TFP)………………….S4

3. Structure of the dual-modality linker (DML), previously reported construct………………….S7

4. Construction and characterization of the modular cys-diabody, **TFP-cDb**……………………S8

5. Radiochemistry……………………………………………………………………………..S9

5.1 General materials and methods…………………………………………………………S9

5.2 Synthesis of [^{18}F]fluoroethoxy TCO……………………………………………..S10

6. Positron Emission Tomography (PET) Imaging……………………………………………..S10

6.1 ^{18}F-Labeling of the modular cys-diabody, **TFP-cDb**, to afford **^{18}F-cDb**……………S10

6.2 Immunoreactivity Measurement …………………………………………………....S10

6.3 Prostate cancer xenograft mouse model (22Rv1-PSCA) …………………………..S11

6.4 *In vivo* microPET/CT imaging……………………………………………………….S11

6.5 ROI analysis and *ex vivo* biodistribution……………………………………………S12

6.6 *Ex vivo* biodistribution of **^{18}F-cDb** in normal mice…………………………………S13

6.7 *Ex vivo* biodistribution of **^{18}F-cDb** in tumor-bearing………………………………..S14

7. Near-Infrared Fluorescence (NIRF) imaging………………………………………………...S15

7.1 Labeling of **TFP-cDb** with sulfoCy5 TCO and characterization **sCy5-cDb**………..S15

7.2 *In vivo* NIRF imaging in tumor-bearing mice………………………………………S15

8. References……………………………………………………………………………………S16

1. Materials and Methods

Chemistry - General Procedures

All chemicals and reagents were purchased from commercial sources and used without further purification. Deuterated solvents were purchased from Cambridge Isotope Laboratories. 1,4,7,10-Tetraazacyclododecane-1,4,7,10-tetraacetic acid mono-N-hydroxysuccinimide ester (DOTA-NHS-ester, catalog # B-280) was purchased from Macrocyclics. Mal-amido-PEG2-NHS (catalog # BP-22156) was purchased from Broadpharm. Sulfo-Cy5-TCO (sc-496985) was purchased from Santa Cruz Biotechnology and used as a solution in DMF (20 mM). 4-(6-methyl-1,2,4,5-tetrazin-3-yl)benzyl amine was synthesized according to a previous literature procedure.[1] [^{18}F]fluoroethoxy TCO was synthesized and formulated according to a previous literature procedure.[2] Unless otherwise noted, reactions were carried out in oven-dried glassware under an atmosphere of argon using anhydrous solvents. Anhydrous solvents were obtained by filtration through activated alumina columns unless indicated otherwise. Solvents used for extractions and chromatography were not anhydrous. Reactions and chromatography fractions were analyzed by thin-layer chromatography (TLC) using Merck precoated silica gel 60 F_{254} glass plates (250 μm) and visualized by ultraviolet irradiation, potassium permanganate stain, and phosphomolybdic acid. Flash column chromatography was performed using E. Merck silica gel 60 (230−400 mesh) with compressed air.

NMR spectra were recorded on a Bruker AV300 (300 MHz for ^{1}H), a Bruker AV500 (500 MHz for ^{1}H; 125 MHz for ^{13}C), and a Bruker AV600 (600 MHz for ^{1}H; 150 MHz for ^{13}C). ^{1}H and ^{13}C chemical shifts are reported in parts per million (ppm) using the residual solvent peak as an internal reference. The coupling constants, *J*, are reported in Hertz (Hz), and the multiplicity are reported as follows: singlet (s), broad singlet (br s), doublet (d), triplet (t), quartet (q) and multiplet (m).

High-resolution mass spectrometry data were collected with a Waters LCT Premier XE time-of-flight instrument controlled by MassLynx 4.1 software or obtained on a Thermo Scientific™ Exactive Mass Spectrometer with DART ID-CUBE. Samples were dissolved in methanol and infused using direct loop injection from a Waters Acquity UPLC into the Multi-Mode Ionization source. HPLC purifications were performed on a Knauer Smartline HPLC system with inline Knauer UV (254 nm) detector. Semi-preprative HPLC was performed using Phenomenex reverse-phase Luna column (10 × 250 mm, 5 µm) with a flow rate of 4 mL/min. Final purity of compounds was determined by analytical HPLC analysis performed with a Phenomenex reverse-phase Luna column (4.6 × 250 mm, 5 µm) with a flow rate of 1 mL/min. Compounds were identified by UV absorbance at 254 nm. All chromatograms were collected by a GinaStar (raytest USA, Inc.; Wilmington, NC, USA) analog to digital converter and GinaStar software (raytest USA, Inc.).

2. Experimental Data

2.1 Synthesis and characterization of the tri-functional platform (TFP)

Intermediates **1a** and **2** were synthesized according to literature procedure.[3]

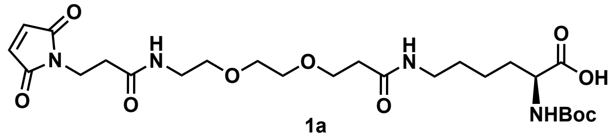

1a

(*S*)-19-((tert-butoxycarbonyl)amino)-1-(2,5-dioxo-2,5-dihydro-1*H*-pyrrol-1-yl)-3,13-dioxo-7,10-dioxa-4,14-diazaicosan-20-oic acid (1a). A 10 mL round bottom flask equipped with a stir bar and filled with argon, was charged with *N*-Boc-L-lysine (61 mg, 0.248 mmol, 1.1 equiv) and dry DMF (1 mL). To this, *N*,*N*-diisopropylethylamine (86 µL, 0.495 mmol, 2.5 equiv) was added and the mixture was stirred at room temperature for 1 h. Malamido-PEG2-NHS (100 mg, 0.23

mmol, 1.0 equiv) then was added and the reaction mixture was stirred at room temperature until full consumption of starting materials was indicated by TLC. DMF was removed under reduced pressure and the crude mixture was purified by flash column chromatography on silica gel (5% MeOH in DCM to 10% MeOH in DCM) to give 100 mg of the desired product as a colorless oil (78% yield). Purified **1a** was stored under argon at -20°C. ^{1}H and ^{13}C NMR spectroscopic data were consistent with previously reported values.[3]

HRMS (ESI) *m/z* calcd for $C_{25}H_{40}N_{4}O_{10}$ $[M + H]^{+}$, 557.2817, found 557.2847.

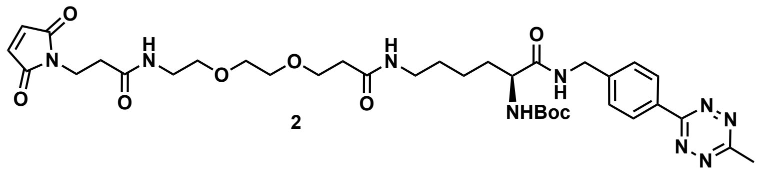

Tert-butyl (*S*)-(22-(2,5-dioxo-2,5-dihydro-1*H*-pyrrol-1-yl)-1-(4-(6-methyl-1,2,4,5-tetrazin-3-yl)phenyl)-3,10,20-trioxo-13,16-dioxa-2,9,19-triazadocosan-4-yl)carbamate (2). To a solution of **1a** (60 mg, 108 µmol, 1.0 equiv) in DMF (1.2 mL) was added HATU (46 mg, 119 µmol, 1.1 equiv) and the mixture was stirred for 10 min at room temperature. To this, a solution of 4-(6-methyl-1,2,4,5-tetrazin-3-yl)benzyl amine[1] (76 mg, 319 µmol, 2.96 equiv) in DMF (1.5 mL) was added and the mixture was stirred for another 20 min. *N*,*N*-diisopropylethylamine (54 µL, 313 µmol, 2.9 equiv) was added and the mixture was stirred at room temperature for 36 hours. DMF was removed under reduced pressure and the crude mixture was purified by flash column chromatography on silica gel (0% to 10% MeOH in DCM) to give 49 mg of the desired product as a deep purple oil (71% yield). Characterization data were consistent with previously reported data.[3]

ESI(+)-MS: calcd. for $C_{35}H_{49}N_{9}O_{9} + H^{+}$, 762.3545; found 762.3546

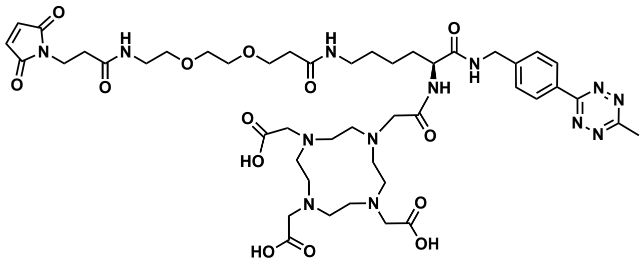

Tri-functional Platform
3

(*S*)-2,2',2''-(10-(22-(2,5-Dioxo-2,5-dihydro-1*H*-pyrrol-1-yl)-4-((4-(6-methyl-1,2,4,5-tetrazin-3-yl)benzyl)carbamoyl)-2,10,20-trioxo-13,16-dioxa-3,9,19-triazadocosyl)-1,4,7,10-tetraazacyclododecane-1,4,7-triyl)triacetic acid (3). To a 4 ml vial containing Boc-protected amine **2** (52.6 mg, 71.1 µmol, 1.0 equiv) was added a solution of HCl/dioxane (4 M, 360 µL) at 0 °C. The reaction mixture was allowed to warm to room temperature and stirred for 6 h. The solvent was removed and the crude residue was subjected to semi-preparative HPLC purification.[3] The fraction containing the product was lyophilized yielding 19 mg of desired compound as a purple solid (40 % yield). Due to instability issues, the intermediate was immediately conjugated to commercial DOTA-NHS ester. To a 1.5 mL Eppendorf tube was added **3** (3.6 mg, 5.32 µmol, 1 equiv) and dry DMF (0.16 mL). To this, triethylamine (7.4 µL, 53.2 µmol, 10 equiv) was added and the mixture was stirred at room temperature for 15 min. 1,4,7,10-Tetraazacyclododecane-1,4,7,10-tetraacetic acid mono-N-hydroxysuccinimide ester (4 mg, 5.32 µmol, 1 equiv) was added and the reaction mixture was stirred at room temperature for 16 hours. DMF was removed under reduced pressure and the residue was subjected to semi-preparative HPLC purification to afford 2.7 mg of the tri-functional platform **3** as a dark purple solid (49% yield).

HRMS (ESI) *m/z* calcd for $C_{46}H_{68}N_{13}O_{14}$ $[M + H]^+$, 1026.5009, found 1026.5046.

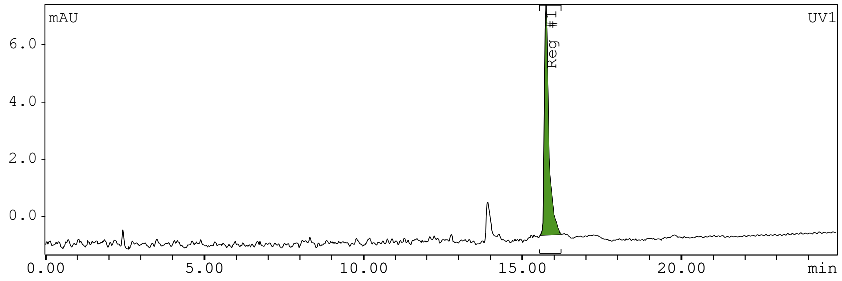

Figure S1. Analytical HPLC trace of purified 3. HPLC mobile phase: 5% acetonitrile in water (both in 0.1% TFA) for 0-5 min, then gradient is increased to 75% acetonitrile in water for 5-25 min.

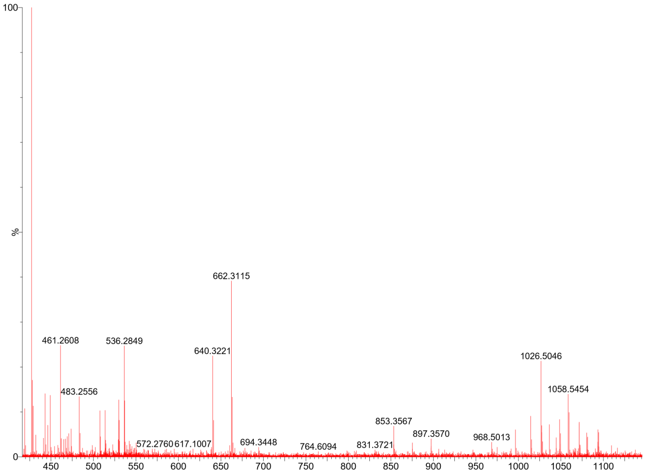

Figure S2. HRMS trace of purified compound **3**.

3. Structure of the dual-modality linker (DML), previously reported construct.

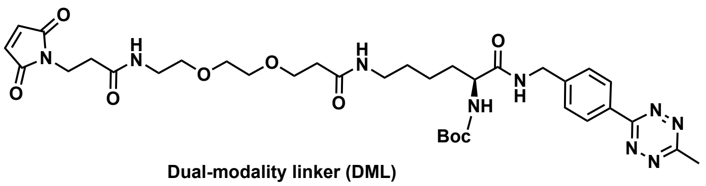

Dual-modality linker (DML)

4. Construction and characterization of the modular cys-diabody, TFP-cDb

Generation and production of the anti-PSCA cys-diabody 2B3 A2 (**cDb**) were described previously.[4] Site-specific conjugation was performed as previously described[3]. Briefly, the protein was dissolved in PBS pH 7.4 and incubated with tris-(2-carboxyethyl)phosphine, hydrochloride (TCEP, 10-fold molar excess) for 30 min at room temperature to reduce the C-terminal cystine disulfides. Following reduction, a solution of **3** in DMF (3- to 10-fold molar excess) was added to the reduced protein and the reaction was incubated for 2 h at room temperature. Excess reagents were separated from the protein conjugate using Micro Bio-Spin size exclusion columns (BioRad) pre-blocked with PBS, 1%FBS. The modular cys-diabody **TFP-cDb** was analyzed by polyacrylamide gel electrophoresis (SDS-PAGE) and size exclusion chromatography (SEC) using a Superdex-75 HR10/30 column in an Äkta purifier (GE Healthcare). The interchain disulfide bond provides the stabilizing force for the cys-diabody dimer which, following reduction, is merely held together by weak non-covalent interactions. Therefore, in SDS-PAGE, the nonreduced anti-PSCA A2 cys-diabody migrates as a 50 kDa dimeric band, whereas the completely reduced protein migrates as a 25 kDa monomeric band. Upon reduction, the free cysteines can undergo a Michael addition with **3**, forming a stable thioether linkage and preventing dimer formation via the interchain disulfide bond. Thus, the monomeric band in **Figure S3** confirms successful conjugation of **3** to afford the modular cys-diabody **TFP-cDb**. To evaluate the purity and integrity of **TFP-cDb**, size exclusion chromatography was conducted (**Figure S3**). Both the unconjugated protein **cDb** and the conjugated protein **TFP-cDb** elute with similar retention times indicating that protein reduction and conjugation to compound **3** did not impede dimer formation.

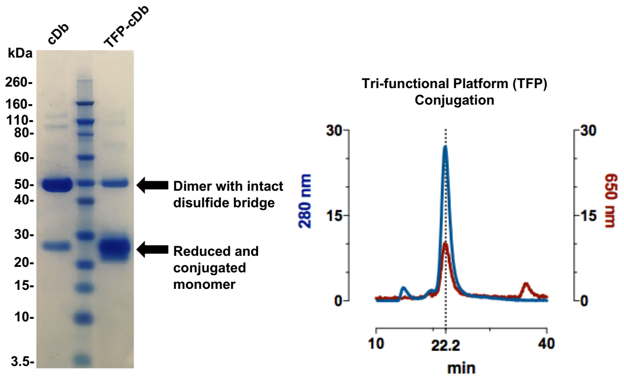

Figure S3. Construction and characterization of the modular cys-diabody, TFP-cDb. SDS-PAGE gel shows purified cys-diabody (cDb lane) and reduced and site-specific conjugated cys-diabody (TFP-cDb lane). Size exclusion chromatography confirms the integrity of the diabody (dimeric conformation) is preserved in the site-specific TFP-cDb conjugate.

5. Radiochemistry

5.1 General materials and methods

No-carrier-added [^{18}F]fluoride was produced by the ^{18}O(p,n)^{18}F nuclear reaction in a Siemens RDS-112 cyclotron at 11 MeV using a 1 mL tantalum target with havar foil. Unless otherwise stated, reagents and solvents were commercially available and used without further purification. HPLC grade acetonitrile and trifluoroacetic acid were purchased from Fisher Scientific. Anhydrous acetonitrile, dimethyl sulfoxide and tetraethylammonium bicarbonate were purchased from Sigma-Aldrich. Sterile product vials were purchased from Hollister-Stier. QMA-light Sep-Paks and tC18 light cartridges were purchased from Waters Corporation. Radio-TLCs were analyzed using a miniGITA* TLC scanner. HPLC purifications were performed on a Knauer Smartline HPLC system with inline Knauer UV (254 nm) detector and gamma-radiation coincidence detector and counter (Bioscan Inc.). Semi-preprative HPLC was performed using

Phenomenex reverse-phase Luna column (10 × 250 mm, 5 μm) with a flow rate of 4 mL/min. Final purity and identity of compounds were determined by analytical HPLC analysis performed with a Phenomenex reverse-phase Luna column (4.6 × 250 mm, 5 μm) with a flow rate of 1 mL/min. All chromatograms were collected by a GinaStar (Raytest) analog to digital converter and GinaStar software.

5.2 Synthesis of [^{18}F]fluoroethoxy TCO

[^{18}F]fluoroethoxy TCO was synthesized with the ELIXYS FLEX/CHEM radiosynthesizer (Sofie Biosciences) and reformulated in 0.5% aq. sodium ascorbate:DMSO solution, 1:1 (v/v) as reported previously.[2]

6. Positron Emission Tomography (PET) Imaging

6.1 ^{18}F-Labeling of the modular cys-diabody, TFP-cDb, to afford ^{18}F-cDb

TFP-cDb was incubated with [^{18}F]fluoroethoxy TCO (37 MBq) at room temperature for 10 minutes to afford the (4 + 2) cycloaddition product, **^{18}F-cDb**. **^{18}F-cDb** was purified using Micro Bio-spin columns. Radiochemical purity and labeling efficiency was determined by ITLC (ITLC strips for monoclonal antibody preparation, Biodex Medical Systems). Samples were spotted at the origin and developed in acetonitrile as the solvent. The ITLC strips were cut in half and gamma counted in a Wizard 3' 1480 Automatic Gamma Counter (Perkin Elmer).

6.2 Immunoreactivity Measurement

The percentage of immunoreactive ^{18}F-cDb was measured by incubating approximately 0.5 ng of radiolabeled protein with excess (30-45 x 10^6) human PSCA-expressing prostate cancer cells

(22Rv1-PSCA) in PBS/1% FBS for 1 h at room temperature. Cells were centrifuged and radioactivity was counted in a gamma counter. The immunoreactive fraction was calculated as the percentage of cell bound activity in the pellet over total activity (pellet plus supernatant).

6.3 Prostate cancer xenograft mouse model (22Rv1-PSCA)

All procedures involving animals were conducted in accordance with the NIH Guide for the Care and Use of Laboratory Animals (USA), under protocols approved by the UCLA Chancellor's Animal Research Committee. Cell lines 22Rv1 and 22Rv1-PSCA were previously described[5] and were cultured in RPMI1640, 10% FBS. Tumors were inoculated subcutaneously ($1x10^6$ cells/100 µL in 1:1 PBS:Matrigel (BD Biosciences)) in the shoulder of male nude mice (8-12 weeks, JAX002019, Jackson Laboratories) as previously described.[6]

6.4 *In vivo* microPET/CT imaging

Approximately 10 µg (1.3-1.85 MBq) of **^{18}F-cDb** was injected into the tail vein of male nude mice with or without s.c. tumors. Mice were anesthetized with 1.5% isoflurane and small animal PET scans (G8 PET/CT, Perkin Elmer) were acquired for 60 min (dynamic scan, n=1) followed by static 10-min scans (n=4) at 2 and 4 hours p.i. CT scans were subsequently acquired for anatomical co-registration and attenuation correction. Images were reconstructed using the Maximum Likelihood Estimation Method (MLEM) algorithm and displayed as whole-body maximum intensity projections/CT overlays (**Figure S4**).

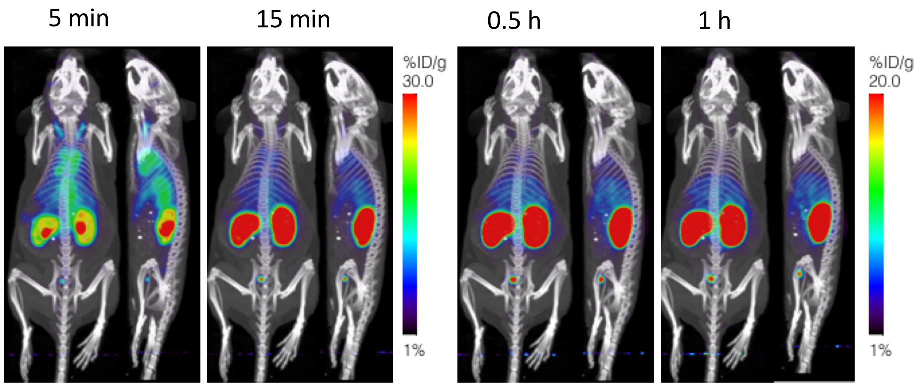

Figure S4. ^{18}F-cDb immunoPET in non-tumor bearing mice. ^{18}F-cDb (1.3 MBq/8.5 µg) was injected i.v. into male nude mice (Nu/J). Representative images acquired during a 60-minute dynamic scan (n=1) are depicted as whole-body MIP/CT overlays.

6.5 ROI analysis and *ex vivo* biodistribution

PET image analysis was performed using AMIDE.[7] Quantitative region of interest (ROI) analysis was performed by converting the mean voxel value to percent injected dose per gram (%ID/g_{ROI}, assuming a tissue density of 1 g/mL) using the decay-corrected injected dose. Following the final scans, mice were euthanized and *ex vivo* biodistribution analysis was performed. Major organs and tissues were dissected, weighed and the radioactivity was measured using a gamma counter. The percent-injected dose per gram of tissue (%ID/g) was calculated using a standard containing 1% of the injected dose. Radioactivity in the kidneys saturated the count-rate capacity of the gamma counter (**Table S1**); **Figure 4** provides a separate determination of kidney activity.

6.6 *Ex vivo* biodistribution of ^{18}F-cDb in normal mice

Organ	Mean ± SEM
Blood	0.07 ± 0.01
Heart	0.13 ± 0.03
Lung	0.17 ± 0.02
Kidney	14.86* ± 0.49
Liver	0.51 ± 0.06
Gallbladder	3.57 ± 0.47
Spleen	0.29 ± 0.06
Stomach	0.10 ± 0.02
Intestine	0.61 ± 0.06
Muscle	0.04 ± 0.00
Bone	0.19 ± 0.02
Prostate + Sem. Vesicles	0.07 ± 0.01
Carcass	0.09 ± 0.01

Table S1. Ex vivo biodistribution of ^{18}F-cDb in normal male nude mice 4 h post-injection. Values are presented as %ID/g Mean ± SEM of n=4. *the maximum count rate of the gamma detector was exceeded when measuring the kidney.

6.7 *Ex vivo* biodistribution of ^{18}F-cDb in tumor-bearing mice

Organ	Mean ± SEM
Blood	0.32 ± 0.01
Positive Tumor	2.03 ± 0.01
Negative Tumor	0.27 ± 0.26
Heart	0.64 ± 0.03
Lung	0.96 ± 0.11
Liver	2.51 ± 0.15
Gallbladder	9.54 ± 4.74
Spleen	1.11 ± 0.06
Kidney	75.58 ± 3.06
Stomach	0.44 ± 0.07
Intestine	3.72 ± 0.21
Muscle	0.33 ± 0.03
Bone	1.13 ± 0.14
Tail	1.75 ± 0.44
Carcass	0.53 ± 0.06
Prostate	1.49 ± 0.58
**Tumor-to-organ ratios**	
Positive Tumor:Blood	6.34
Positive:Negative Tumor	7.52
Positive Tumor:Muscle	6.15

Table S2. Ex vivo biodistribution of ^{18}F-cDb in male nude mice bearing PSCA xenografts following PET quantification (%ID/g) 4 h post-injection.

7. Near-Infrared Fluorescence (NIRF) Imaging

7.1 Labeling of TFP-cDb with sulfoCy5 TCO and characterization of sCy5-cDb.

TFP-cDb was incubated with sCy5-TCO at room temperature for 10 minutes to afford the (4 + 2) cycloaddition product, **sCy5-cDb**. Following procedures described above, excess label was removed using Micro Bio-spin columns and **sCy5-cDb** was analyzed by SDS-PAGE and SEC (**Figure S5**). Dye and protein concentrations were measured using a spectrophotometer at 650 nm and 280 nm, respectively and the dye:protein ratio was determined to be 1.1.

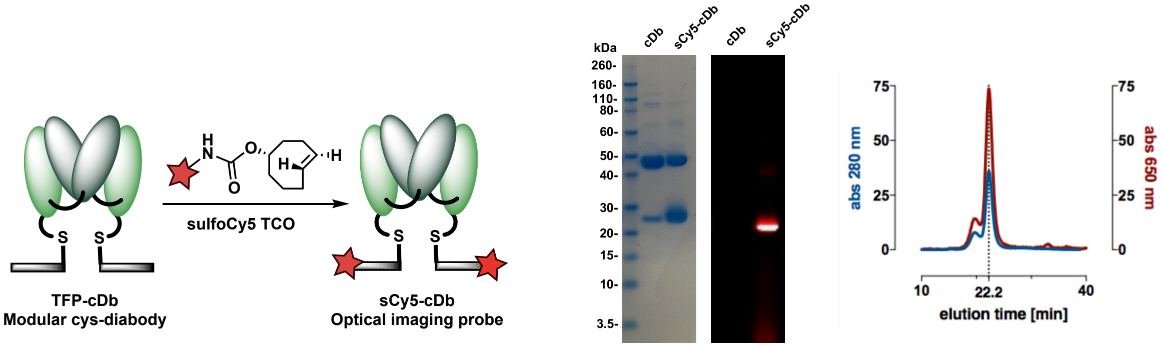

Figure S5. Construction and characterization of sCy5-cDb. Site-specific conjugation of purified cDb with TFP followed by (4+2) cycloaddition reaction with sCy5-TCO to afford the fluorescently labeled diabody, sCy5-cDb. Size exclusion chromatography was conducted to evaluate purity and integrity of sCy5-cDb. Successful labeling with sCy5 was achieved without interfering with the dimeric conformation of the diabody.

7.2 *In vivo* NIRF imaging in tumor-bearing mice

Optical imaging was conducted post mortem with the skin removed or of dissected tumors and organs *ex vivo* using the IVIS Lumina II system (PerkinElmer) with settings: 675 ex/694 em (Cy5.5) and 5 sec exposure time. Living Image Software (IVIS Imaging Systems, PerkinElmer) was used to display fluorescence/visible light overlays.

8. References

[1] J. Yang, M. R. Karver, W. Li, S. Sahu, N. K. Devaraj, *Angew. Chem. Int. Ed.* **2012**, *51*, 5222-5225.

[2] J. Collins, C. M. Waldmann, C. Drake, R. Slavik, N. S. Ha, M. Sergeev, M. Lazari, B. Shen, F. T. Chin, M. Moore, S. Sadeghi, M. E. Phelps, J. M. Murphy, R. M. van Dam, *Proc. Natl. Acad. Sci. U.S.A.* **2017**, *114*, 11309-11314.

[3] K. A. Zettlitz, C. M. Waldmann, W.-T. K. Tsai, R. Tavaré, J. Collins, J. M. Murphy, A. M. Wu, *J. Nucl. Med.* **2019**, 10.2967/jnumed.2118.223560.

[4] a) E. J. Lepin, J. V. Leyton, Y. Zhou, T. Olafsen, F. B. Salazar, K. E. McCabe, S. Hahm, J. D. Marks, R. E. Reiter, A. M. Wu, *Eur J Nucl Med Mol Imaging* **2010**, *37*, 1529-1538; b) G. A. Sonn, A. S. Behesnilian, Z. K. Jiang, K. A. Zettlitz, E. J. Lepin, L. A. Bentolila, S. M. Knowles, D. Lawrence, A. M. Wu, R. E. Reiter, *Clin Cancer Res* **2016**, *22*, 1403-1412.

[5] D. C. Saffran, A. B. Raitano, R. S. Hubert, O. N. Witte, R. E. Reiter, A. Jakobovits, *Proc Natl Acad Sci U S A* **2001**, *98*, 2658-2663.

[6] K. A. Zettlitz, W. K. Tsai, S. M. Knowles, N. Kobayashi, T. R. Donahue, R. E. Reiter, A. M. Wu, *J Nucl Med* **2018**, *59*, 1398-1405.

[7] A. M. Loening, S. S. Gambhir, *Mol Imaging* **2003**, *2*, 131-137.
